# High-sensitivity CRP may be a marker of HDL dysfunction and remodeling in patients with acute coronary syndrome

**DOI:** 10.1038/s41598-021-90638-0

**Published:** 2021-06-01

**Authors:** Xiaoyu Tang, Ling Mao, Jin Chen, Tianhua Zhang, Shuwei Weng, Xin Guo, Jie Kuang, Bilian Yu, Daoquan Peng

**Affiliations:** Department of Cardiovascular Medicine, Research Institute of Blood Lipids and Atherosclerosis, The Second Xiangya Hospital of Central South University, No. 139 Middle Renmin Road, Changsha, 410011 Hunan China

**Keywords:** Biochemistry, Cardiology

## Abstract

In patients with coronary artery disease (CAD), further increasing the level of high-density lipoprotein (HDL) cholesterol (HDL-C) as an add-on to statins cannot reduce cardiovascular risk. And it has been reported that HDL functional metric—cholesterol efflux capacity (CEC) may be a better predictor of CAD risk than HDL-C. CEC measurement is time-consuming and not applicable in clinical settings. Thus, it is meaningful to explore an easily acquired index for evaluating CEC. Thirty-six CAD patients and sixty-one non-CAD controls were enrolled in this cross-sectional study. All CAD patients had acute coronary syndrome (ACS). CEC was measured using a [^3^H] cholesterol loading Raw 264.7 cell model with apolipoprotein B-depleted plasma (a surrogate for HDL). Proton nuclear magnetic resonance (NMR) spectroscopy was used to assess HDL components and subclass distribution. CEC was significantly impaired in CAD patients (11.9 ± 2.3%) compared to controls (13.0 ± 2.2%, *p* = 0.022). In control group, CEC was positively correlated with enzymatically measured HDL-C levels (r = 0.358, *p* = 0.006) or with NMR-determined HDL-C levels (NMR-HDL-C, r = 0.416, *p* = 0.001). However, in CAD group, there was no significant correlation between CEC and HDL-C (r = 0.216, *p* = 0.206) or NMR-HDL-C (r = 0.065, *p* = 0.708). Instead, we found that the level of high-sensitivity C-reactive protein (hsCRP) was inversely associated with CEC (r = − 0.351, *p* = 0.036). Multiple regression analysis showed that the hsCRP level was associated with CEC after adjusting other cardiovascular risk factors and HDL-C, although the association would not reach significance if adjusting for multiple testing. NMR spectroscopy showed that HDL particles shifted to larger ones in patients with high hsCRP levels, and this phenomenon was accompanied by decreased CEC. In patients with CAD, the level of HDL-C cannot reflect HDL function. The impaired correlation between HDL-C and CEC is possibly due to an inflammation-induced HDL subclass remodeling. These hypothesis-generating data suggest that hsCRP levels, a marker of acute inflammation, may associate with HDL dysfunction in ACS subjects. Due to the design limited to be correlative in nature, not permitting causal inference and a larger, strictly designed study is still needed.

## Introduction

Over the last few decades, epidemiological studies have confirmed a strong and inverse relationship between the level of high-density lipoprotein (HDL) cholesterol (HDL-C) and the risk of coronary artery disease (CAD)^1^. However, the role of HDL-C has been challenged by the failure of HDL-C raising trials using niacin or cholesteryl ester transfer protein (CETP) inhibitors^2,3^. In addition, genetically increased HDL-C does not necessarily translate to a decreased risk of myocardial infarction^4^. Even worse, higher HDL-C levels secondary to *SCARB1* gene mutations lead to an increased risk of CAD^5^. A recent epidemiological study has also revealed that extremely high HDL-C levels are associated with increased CAD mortality^6^. These results highlight the potential limitations of using HDL-C levels, a static mass-based parameter, to assess the risk of CAD, and call for investigations on more robust HDL functional markers for evaluating cardiovascular risks.

HDL exerts favorable effects against atherosclerosis, primarily by reverse cholesterol transport (RCT)^7^. Cholesterol efflux capacity (CEC), a metric reflecting the ability of HDL as a cellular cholesterol acceptor, has been demonstrated to be inversely associated with subclinical atherosclerosis^8,9^, the incidence of cardiovascular events^10–12^, and prognosis of CAD^13,14^, and is now considered a reliable CAD marker^10^.

The measurement of CEC requires radiolabeled cholesterol and cultured cells, which is time-consuming and not applicable in clinical settings. It has been observed that CEC was positively correlated with HDL-C levels in healthy populations^8,11,12,15^. However, in patients with CAD, this relationship was inconsistent in different studies. Data from Khera et al.^8^ and Shao et al.^16^ showed that the correlation coefficients between HDL-C and CEC were 0.51 (*p* < 0.0001) and 0.31 (*p* < 0.05), respectively. In contrast, two other studies showed that the correlation was weak^17^ or even inexistent^13^.

The unstable relationship between HDL-C and CEC may be subjected to dynamic changes in the components of HDL subclasses. Inflammation has been a well-established factor that affects HDL components and subclass distribution^18,19^, and patients with inflammatory connective tissue disease always present a decreased CEC^20^, suggesting inflammatory markers may serve as surrogate parameters for HDL dysfunction.

Proton nuclear magnetic resonance (NMR) spectroscopy is an emerging technique that can provide a fine-grained snapshot of a person’s lipid metabolism. Using NMR-determined HDL subclasses can improve the mortality risk discrimination in the cardiac catheterization cohort^21^ and predict the prognosis of patients with pulmonary arterial hypertension^22^. This study used NMR spectroscopy to provide more detailed information about the components of HDL and HDL subclasses. By simultaneously examining the level of high-sensitivity C-reactive protein (hsCRP), a sensitive marker of systemic inflammation, and HDL functional marker CEC, we intended to clarify the relationship between HDL-C and HDL subclasses and CEC as well as between hsCRP and CEC in patients with CAD.

## Subjects and methods

### Study population

We used samples and data from a trial registered at Chinese Clinical Trial Registry as ChiCTR1900020873. The object of the trial was to characterize lipid profiles by NMR spectroscopy at fasting and non-fasting states in CAD and non-CAD subjects, which has been reported^23^. From June 2018 to December 2018, we consecutively recruited 50 CAD patients, who were 18–80 years old, presented with myocardial ischemia symptoms and suspected with CAD in the Department of Cardiovascular Medicine of the Second Xiangya Hospital of Central South University. Patients during the gestational phase, under hormone therapy, taking anti-inflammatory drugs were not included. All these patients underwent coronary angiography, and the diagnosis was confirmed by a coronary angiography showing ≥ 50% stenosis in at least one main coronary artery. Forty-two patients were diagnosed with CAD. Patients with significant hematologic disorders, infectious or inflammatory disease, various tumors, severe liver and/ or renal insufficiency, severe uncontrolled diabetes or hypertension, and alcohol use or intensive exercise in the week before enrollment were excluded, and 36 CAD patients were finally enrolled. We also enrolled eighty apparently healthy subjects in the same period, and they were free from atherosclerotic disorders as confirmed by coronary angiography or coronary computed tomography angiography. The excluding criteria was the same as CAD patients, and 61 non-CAD subjects were finally enrolled. Written informed consent was obtained from all the individuals. The research related to human use complied with all the relevant national regulations, institutional policies and followed the tenets of the Helsinki Declaration, and has been approved by the Medical Ethics Committee of the Second Xiangya Hospital of Central South University. This trial was registered at the Chinese Clinical Trial Registry as ChiCTR1900020873.

### Clinical and biochemical measurements

Demographic information, such as height, weight, blood pressure, and heart rate were measured and recorded. Fasting blood samples were collected the morning after admission. Routine blood, urine, and lipid profiles, including triglycerides (TG), total cholesterol (TC), low-density lipoprotein cholesterol (LDL-C), and HDL-C, were analyzed via the enzymatic method. hsCRP was measured with a latex particle, enhanced immunoturbidimetric assay. Cardiac troponin T was measured using a high-sensitivity assay. For the subsequent experiments, fresh plasma was aliquoted and stored at − 80 °C.

### ApoB-depleted plasma preparation

The plasma samples were thawed in a refrigerator at 4 °C before the experiment. According to the protocol of the previous experiment^24^, 540 µL of heparin sodium solution (280 mg/mL, Aladdin, China) and 10 mL of a manganese chloride solution (1.06 mol/L, Aladdin, China) were mixed. Plasma was incubated for 30 min at 4 °C with a mixed solution (10:1 vol/vol) and then centrifuged at 1500 × *g* for 30 min. The supernatant was collected, and if it was still turbid (especially samples with a high concentration of triglycerides), plasma was centrifuged again at 12,000 × *g* for 10 min, and the lower liquid fraction was recovered for the next procedure. Compared to conventional ultracentrifugation, this precipitation method is simpler and more efficient for HDL isolation^8^. Previous studies have revealed that heparin sodium/manganese chloride precipitation had a minor effect on HDL distribution^25^.

### Measurement of Cholesterol efflux capacity

The cholesterol efflux assay was performed according to established procedures^8,12^. In brief, murine Raw 264.7 macrophages (ATCC, USA) were grown in the Dulbecco’s Modified Eagle Medium (DMEM, Gibco, USA), supplemented with 10% fetal bovine serum (Gibco, USA). Macrophages were plated on 48-well plates (300,000 cells/well). Subsequently, cells were loaded with 25 µg/mL acetylated LDL (Peking Union-Biology Co., Ltd, China) and 1 μCi/mL [^3^H] cholesterol (PerkinElmer, USA) for 24 h. The macrophages were then washed with PBS (Gibco, USA). To upregulate the expression of ATP-binding cassette transporter A1 (ABCA1), cells were stimulated for 24 h with serum-free DMEM containing 0.3 mmol/L 8-Bromoadenosine 3',5'-cyclic monophosphate (Sigma, USA), then washed with PBS again and incubated with 2.8% (vol/vol) apoB-depleted plasma diluted in the medium for 6 h. All steps were performed in the presence of 2 μg/mL of the acyl-coenzyme A: cholesterol acyltransferase (ACAT) inhibitor Sandoz 58–035 (Sigma, USA). The supernatant was collected and centrifuged to remove cellular debris. Cells on the plate were washed with PBS again and then incubated with a 0.1 mol/L NaOH solution for 30 min for cell lysis. The radioactivity within the supernatant and cells was determined by using liquid scintillation counting (PerkinElmer, USA). CEC was calculated using the following equation: CEC (%) = ^3^H media/ (^3^H media + ^3^H cells) × 100. All efflux experiments were performed in triplicate for each sample. Each plate contained blank control and positive control (50 μg/mL HDL, purchased from Peking Union-Biology Co. Ltd). A standard sample (pooled plasma from 20 individuals) was used to correct the inter-assay error.

### Nuclear magnetic resonance spectroscopy

The total plasma apolipoprotein A-I (apoA-I)-rich lipoprotein and 30 discrete HDL-related lipoproteins were measured by NMR spectroscopy at ProteinT Biotechnology Co., Ltd (Tianjin, China) by Bruker 600 MHz NMR spectrometer. Details for the NMR experimental condition were provided in Supplementary Table 6. The spectra were normalized to the same quantitative scale using Bruker’s QuantRef manager within TopSpin which is based on the PULCON method; hence, the spectral intensity is normalized to proton concentration in units of millimoles per liter^26^. The lipoprotein-distribution-prediction method selected for the analysis was the commercial Bruker IVDr Lipoprotein Subclass Analysis (B.I.-LISA) method as previously described^22,27^, which used a PLS-2 regression model as the algorithm for spectral deconvolution^28^. HDL-related lipoproteins were classified into four subclasses, labeled numerically according to decreasing size and increasing density. The HDL1 subclass was the largest, while the HDL4 subclass was the smallest. This method showed exceptional reproducibility with inter coefficient variation of 1.39–2.62%. In the context, HDL-C determined by NMR was described as NMR-HDL-C.

### Statistical analysis

Statistical analysis was performed using the Statistical Package for Social Sciences (SPSS) version 25.0 (IBM, USA, https://www.ibm.com/analytics/spss-statistics-software). All images were made by GraphPad Prism version 8.0 (GraphPad Prism Inc, USA, https://www.graphpad.com/scientific-software/prism/). Normally distributed continuous data have been expressed as mean ± standard deviation. Skewed distributed continuous data have been described as medians with interquartile ranges and were logarithmically transformed when necessary. Comparisons between categorical data were performed with the chi-square test, while continuous variables were assessed by t-test (for normal distribution) or nonparametric tests (for skewed distribution). The Pearson’s correlation analysis was used to evaluate the associations between variables. Multiple linear regression analysis was performed to determine the variables with independent association with CEC. In the correlation and regression analysis, a two-tailed p-value < 0.05 was considered statistically significant.

## Results

### Patient characteristics

All CAD patients had acute coronary syndrome (ACS), including 4 ST-segment elevated myocardial infarction (MI), 14 non-ST-segment elevated MI, and 18 unstable angina. The demographic and biochemical characteristics of the subjects have been shown in Table 1. The subjects in the CAD group were older, with a higher percentage of the male sex, diabetes, hypertension, statin use, and current smoking than subjects in the non-CAD group. Concentrations of TC, HDL-C, and LDL-C were lower, but serum hsCRP was significantly higher in CAD patients than in the non-CAD controls (1.76 [0.88–4.05] vs. 0.91 [0.32–1.87], *p* = 0.004). The median hsTnT level in CAD patients was 0.0178 µg/L (0.0086–0.1667). Other parameters showed no statistically significant differences between the two groups. The interval from symptoms onset to admission for CAD patients has been presented in Supplementary Table 1. Twenty-nine (80.6%) of patients with CAD were admitted to the hospital more than seven days after event onset, and 15 (41.7%) patients were admitted after more than one month of event onset.

**Table 1:** Baseline characteristics of the non-CAD (n = 61) group and the CAD (n = 36) group.

	Non-CAD	CAD
Age, y	46.8 ± 13.3	60.8 ± 8.6**
Male sex, n (%)	27 (44.3)	29 (80.6)**
BMI, kg/m^2^	23.9 ± 3.8	24.8 ± 3.1
Diabetes, n (%)	3 (4.9)	12 (33.3)**
Hypertension, n (%)	16 (26.2)	17 (47.2)*
Statin use, n (%)	4 (6.6)	27 (75.0)**
Current smoking, n (%)	11 (18.0)	15 (41.7)*
Drinking, n (%)	9 (14.8)	7 (19.4)
TG, mg/dL	117.80 (90.34–191.75)	165.18 (105.62–229.40)
TC, mg/dL	170.01 ± 31.73	148.17 ± 36.89*
HDL-C, mg/dL	44.82 ± 10.37	36.04 ± 8.94**
LDL-C, mg/dL	105.98 ± 26.78	89.74 ± 31.58*
hsCRP, mg/L	0.91 (0.32–1.87)	1.76 (0.88–4.05)**
hsTnT, µg/L	NA	0.0178 (0.0086–0.1667)

CAD, coronary artery disease; BMI, body mass index; TG, triglycerides; TC, total cholesterol; LDL-C, low-density lipoprotein cholesterol; hsCRP, high-sensitivity C-reactive protein; hsTnT, high-sensitivity Troponin T; NA, not applicable. *p < 0.05, **p < 0.01.

### Correlation between cholesterol efflux capacity and HDL-C levels

**Figure 1:**
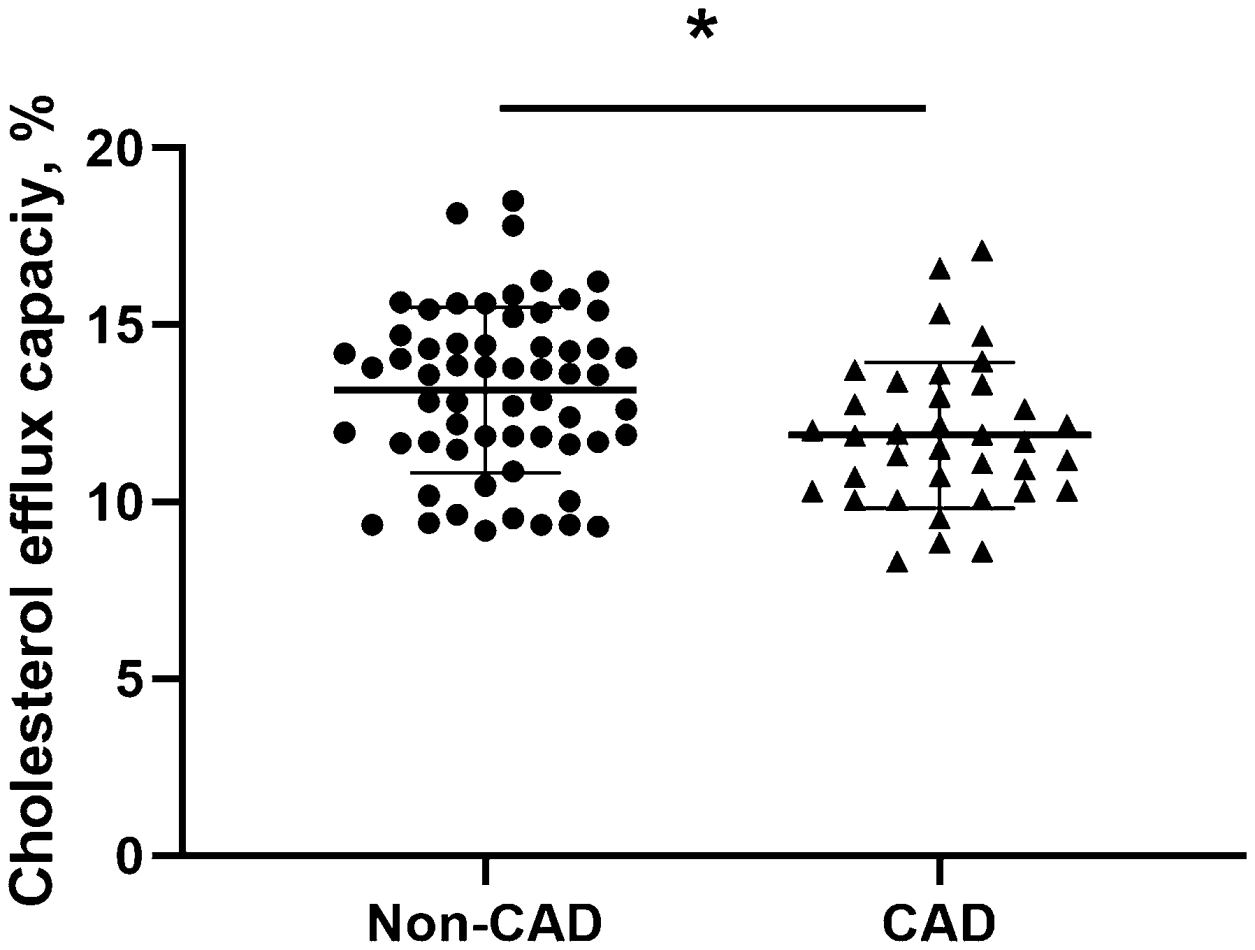
Comparison of CEC between non-CAD controls (n = 61) and CAD patients (n = 36). CEC, cholesterol efflux capacity; CAD, coronary artery disease; *p < 0.05. Data analysis was performed using SPSS version 25.0 (IBM, USA, https://www.ibm.com/analytics/spss-statistics-software) and the figure was made by GraphPad Prism version 8.0 (GraphPad Prism Inc, USA, https://www.graphpad.com/scientific-software/prism/).

**Figure 2:**
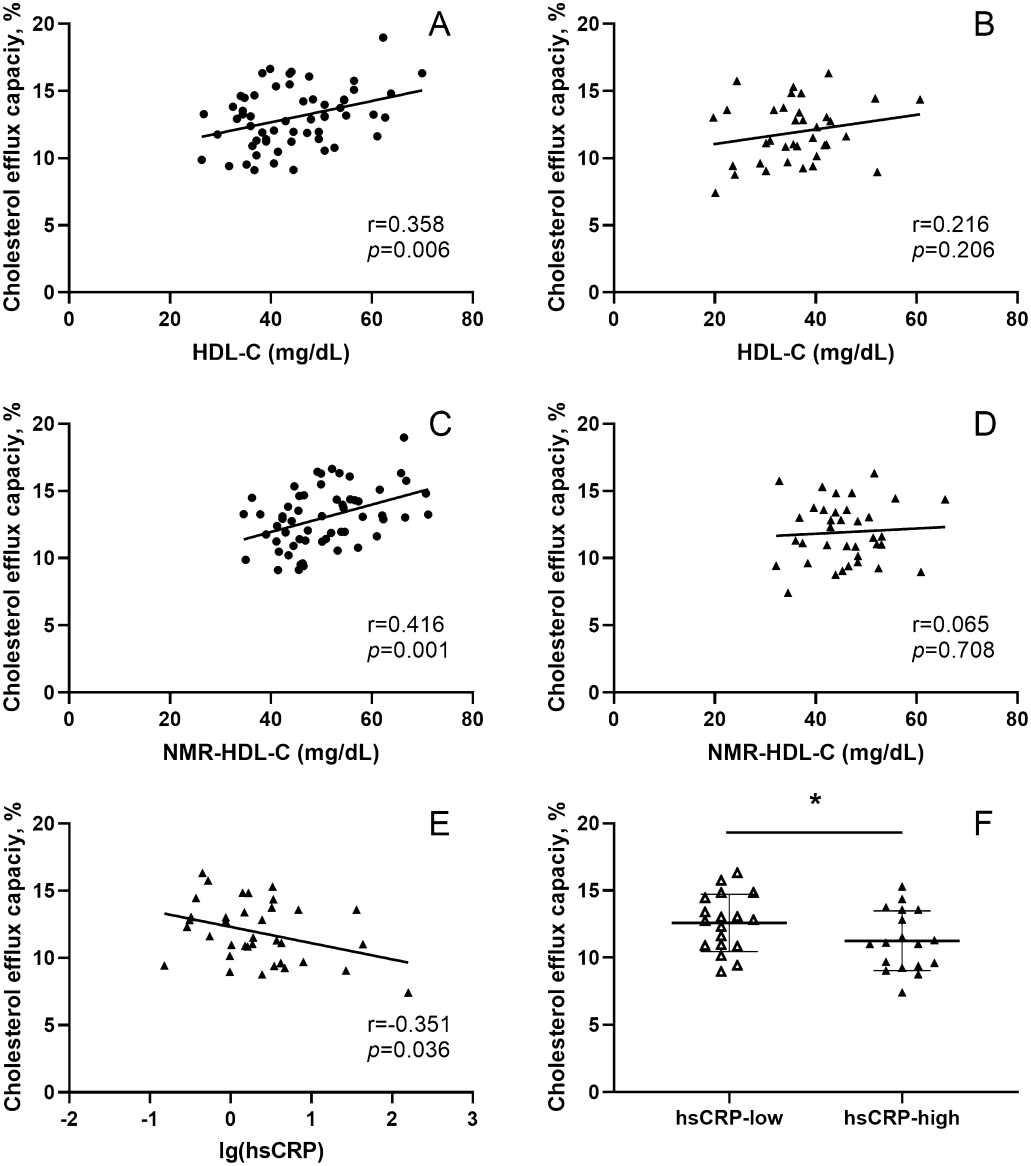
Correlation between CEC and HDL-C in (**A**) non-CAD controls and (**B**) CAD patients. Correlation between CEC and NMR-HDL-C in (**C**) non-CAD controls and (**D**) CAD patients. (**E**) Correlation between hsCRP levels (log-transformed) and CEC in CAD patients (n = 36); (**F**) Comparison of CEC between hsCRP-low CAD patients (hsCRP < 1.75 mg/L, n = 18) and hsCRP-high CAD patients (hsCRP ≥ 1.75 mg/L, n = 18). CEC, cholesterol efflux capacity; CAD, coronary artery disease; NMR-HDL-C, NMR measured high-density lipoprotein cholesterol; hsCRP, high-sensitivity C-reactive protein; CEC, cholesterol efflux capacity; CAD, coronary artery disease; * *p* < 0.05. Statistical analysis was performed using SPSS version 25.0 (IBM, USA, https://www.ibm.com/analytics/spss-statistics-software) and the figure was made by GraphPad Prism version 8.0 (GraphPad Prism Inc, USA, https://www.graphpad.com/scientific-software/prism/).

The CEC of the standard sample on each 48-well plate has been shown in Supplementary Fig. 1. The intra- and inter-assay coefficients of variation were 5.7% and 4.8%, respectively, which was comparable to previous studies^8,29^. CEC in CAD group was significantly lower compared to the non-CAD group (11.9 ± 2.3% vs. 13.0 ± 2.2%, *p* = 0.022, Fig. 1). Correlation analysis showed that HDL-C was positively correlated with CEC in the non-CAD group (r = 0.358, *p* = 0.006, Fig. 2A), while there was no significant correlation in the CAD group (r = 0.216, *p* = 0.206, Fig. 2B).

We also measured the level of HDL-C and other HDL-related lipoproteins using NMR spectroscopy (Supplementary Table 2). Consistent with the results of enzymatic methods, NMR-HDL-C was positively correlated with CEC in non-CAD controls (r = 0.416, *p* = 0.001, Fig. 2C). However, in CAD patients, there was no correlation between NMR-HDL-C and CEC (r = 0.065, *p* = 0.708, Fig. 2D).

In the univariate analysis, as shown in Table 2, CEC was positively correlated with the levels of total plasma apolipoprotein A-I (r = 0.369, *p* = 0.004), HDL-phospholipids (r = 0.338, *p* = 0.009), HDL-free cholesterol (r = 0.282, *p* = 0.032), HDL-apoA-I (r = 0.400, *p* = 0.002), and HDL-apoA-II (r = 0.340, *p* = 0.009) in the non-CAD group. In the CAD group, however, these parameters showed no correlation with CEC.

**Table 2:** Pearson’s correlation analysis between cholesterol efflux capacity and NMR-determined total plasma apolipoprotein A-I-rich lipoprotein and HDL-related lipoproteins in the non-CAD (n = 61) group and the CAD (n = 36) group.

	Non-CAD		CAD	
	r	*p*	r	*p*
Total plasma apoA-I	0.369	**0.004**	0.086	0.618
Total-HDL-triglycerides	0.014	0.918	− 0.190	0.267
Total-HDL-phospholipids	0.338	**0.009**	− 0.028	0.871
Total-HDL-free cholesterol	0.282	**0.032**	0.228	0.182
Total-HDL-apoA-I	0.400	**0.002**	0.070	0.687
Total-HDL-apoA-II	0.340	**0.009**	− 0.071	0.681
HDL1-cholesterol	0.285	**0.030**	− 0.159	0.354
HDL1-triglycerides	0.116	0.384	− 0.230	0.178
HDL1-phospholipids	0.231	0.081	− 0.194	0.256
HDL1-free cholesterol	0.246	0.063	0.089	0.604
HDL1-apoA-I	0.223	0.092	− 0.179	0.297
HDL1-apoA-II	0.165	0.217	− 0.258	0.128
HDL2-cholesterol	0.289	**0.028**	− 0.116	0.499
HDL2-triglycerides	0.035	0.795	− 0.220	0.197
HDL2-phospholipids	0.208	0.116	− 0.209	0.222
HDL2-free cholesterol	0.181	0.173	0.044	0.798
HDL2-apoA-I	0.221	0.096	− 0.198	0.247
HDL2-apoA-II	0.137	0.305	− 0.233	0.171
HDL3-cholesterol	0.319	**0.015**	0.015	0.933
HDL3-triglycerides	0.021	0.878	− 0.205	0.231
HDL3-phospholipids	0.236	0.074	− 0.091	0.600
HDL3-free cholesterol	0.210	0.113	0.112	0.516
HDL3-apoA-I	0.246	0.063	− 0.085	0.622
HDL3- apoA-II	0.155	0.246	− 0.182	0.287
HDL4-cholesterol	0.224	0.091	0.242	0.155
HDL4-triglycerides	0.077	0.566	0.008	0.961
HDL4-phospholipids	0.206	0.120	0.216	0.205
HDL4- free cholesterol	0.194	0.145	0.260	0.126
HDL4-apoA-I	0.238	0.072	0.250	0.141
HDL4-apoA-II	0.182	0.171	0.223	0.192

CEC, cholesterol efflux capacity; NMR, nuclear magnetic resonance; HDL, high-density lipoprotein; CAD, coronary artery disease; ApoA-I, apolipoprotein A-I; ApoA-II, apolipoprotein A-II. Boldface type emphasizes significant changes.

### In the CAD group, cholesterol efflux capacity was negatively correlated with the hsCRP level

To investigate which factor determined HDL-mediated CEC in CAD patients, univariate analysis was performed. There was no correlation between CEC and age, body mass index, severity of coronary stenosis (expressed as the Gensini score), hsTnT, or the serum levels of TG, TC, and LDL-C (see Supplementary Table 3). However, we found that CEC was negatively correlated with the hsCRP level (r = − 0.351, *p* = 0.036, Fig. 2E). Then, we divided 36 CAD patients into hsCRP-low (n = 18) and hsCRP-high (n = 18) groups by using the median hsCRP level value (1.75 mg/L) as the criterion (the values of hsCRP in all patients have been shown in Supplementary Fig. 2). Except for hsTnT, the baseline characteristics, the concentrations of total HDL lipids and apolipoproteins were comparable in two groups (see Supplementary Table 4). Nonetheless, CEC was significantly lower in the hsCRP-high group than in the hsCRP-low group (11.3 ± 2.2% vs. 12.6 ± 2.1%, *p* = 0.038, Fig. 2F).

### In CAD patients, HDL particles underwent extensive remodeling with a high level of hsCRP

**Figure 3:**
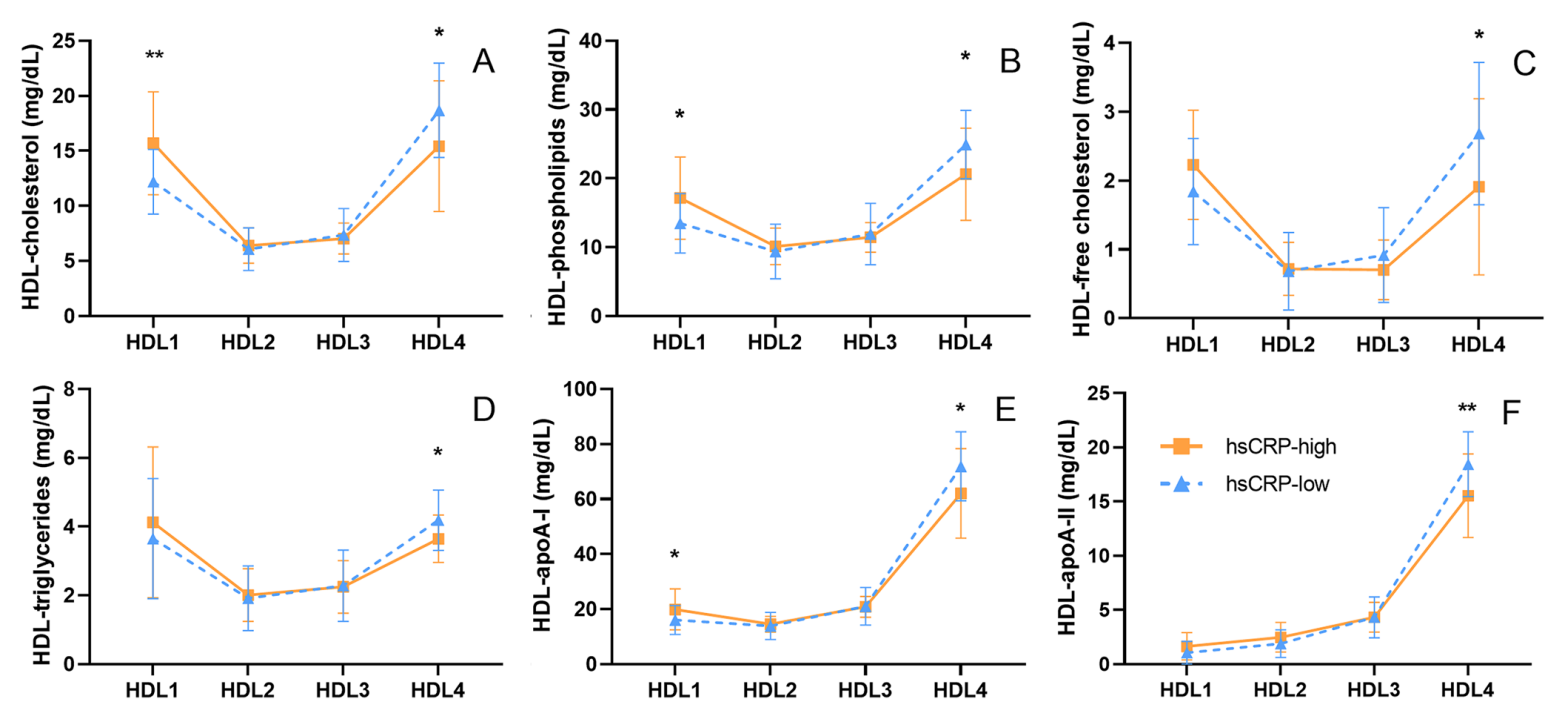
Comparison of 24 HDL-subclass related lipoproteins between hsCRP-low CAD patients (hsCRP < 1.75 mg/L, n = 18) and hsCRP-high CAD patients (hsCRP ≥ 1.75 mg/L, n = 18). HDL subclasses were labeled numerically according to decreasing size and increasing density. HDL, high-density lipoprotein; apoA-I, apolipoprotein A-I; apoA-II, apolipoprotein A-II. Data have been expressed as mean ± SD; *p < 0.05, **p < 0.01. Statistical analysis was performed using SPSS version 25.0 (IBM, USA, https://www.ibm.com/analytics/spss-statistics-software) and the figure was made by GraphPad Prism version 8.0 (GraphPad Prism Inc, USA, https://www.graphpad.com/scientific-software/prism/).

To explore why CEC reduced despite total HDL lipids and major apolipoproteins remained unchanged between the hsCRP-high and the hsCRP-low group, we compared HDL subclass distribution in the two groups. In the hsCRP-high group, the concentrations of lipids and major apolipoproteins in the largest HDL subclass (HDL1) were significantly higher, while those in the smallest HDL subclass (HDL4) were significantly lower than those in the hsCRP-low group (Fig. 3). Further analysis showed that the levels of lipids and major apolipoproteins in HDL1 were positively correlated with the level of hsCRP, while in HDL4, there was a negative correlation (see Supplementary Table 5).

### Multiple linear regression analysis of cholesterol efflux capacity in patients with CAD

To further investigate the relationship between hsCRP and CEC, multiple linear regression analysis was performed. Cardiovascular risk factors including age, sex, LDL-C, diabetes, current smoking, body mass index, TG (log-transformed) and hsTnT (log-transformed) were included as covariates. Adjustments were made for HDL-C, NMR-HDL-C, HDL1-cholesterol, and HDL4-cholesterol. Results showed that hsCRP was associated with HDL-mediated CEC (without Bonferroni correction to adjust the p-value.), regardless of conventional CAD risk factors, HDL-C, and HDL subclasses (Table 3).

**Table 3:** Multiple linear regression analysis between hsCRP and cholesterol efflux capacity in CAD patients (n = 36).

Linear-Regression Covariates*	Beta Coefficient per 1-SD increase in lg(hsCRP) (95% CI)	*p*
Cardiovascular risk factors	− 0.019 (− 0.035 to − 0.004)	**0.016**
Cardiovascular risk factors, HDL-C	− 0.018 (− 0.035 to − 0.002)	**0.027**
Cardiovascular risk factors, NMR-HDL-C	− 0.019 (− 0.035 to − 0.004)	**0.019**
Cardiovascular risk factors, HDL1-cholesterol	− 0.020 (− 0.038 to − 0.002)	**0.032**
Cardiovascular risk factors, HDL4-cholesterol	− 0.019 (− 0.035 to − 0.003)	**0.023**

Cardiovascular risk factors included: age, male sex, LDL-C, diabetes, current smoking, body mass index, triglycerides (log-transformed). hsTnT (log-transformed); hsCRP, high-sensitivity C-reactive protein; CAD, coronary artery disease; NMR-HDL-C, NMR-determined high-density lipoprotein cholesterol. Boldface type emphasizes significant changes.

## Discussion

HDL has been recognized as a traditional protective factor against atherosclerosis^1^. However, subsequent attempts for drug therapies that aim to raise HDL-C levels with niacin^3^ or CETP inhibitor^2^ have both been in vain. HDL-mediated RCT is the key protective function of HDL. CEC, a metric defined by ex vivo experiments that reflects the first and rate-limiting step of RCT, has been demonstrated to be more valuable than HDL-C in predicting CAD risks^8–11,13,14^. However, CEC is susceptible to impairment in many disease states.

Our results showed that compared to non-CAD controls, CEC was lower in CAD patients and was not significantly related to HDL-C. The reduced CEC in CAD patients has already been well described in a high-quality study^8^, but the correlation between HDL-C and CEC was inconsistent in previous reports. For example, the correlation coefficient between HDL-C and CEC was 0.51 (*p* < 0.0001) in the study by Khera et al.^8^ (combining 442 CAD patients and 351 controls) but it was − 0.09 in the study by Zhang et al. (313 CAD patients)^13^. The discordance could result from the different study populations, as most of the patients in Zhang’s study and our study had ACS, while Khera’s study excluded ACS patients. In addition to CAD, the CEC of HDL is also impaired in other diseases, such as acute inflammation^18^, end-stage renal disease^9^, type 1^30^ and type 2 diabetes^11^ or other autoimmune diseases^20^.

We observed that accompanied by decreased CEC, the hsCRP level was inversely correlated with CEC in CAD patients, although the correlation would not reach significance after adjusting for multiple testing. This phenomenon was quite similar to Vaisar’s results^18^. Vaisar et al. found that humans with acute inflammation induced by endotoxin had impaired CEC. HDL proteomic analyses revealed that under that circumstance, the content of serum amyloid A1(SAA1) and SAA2 in HDL was significantly increased, and CEC was inversely correlated with HDL SAA1/SAA2 levels. Although not fully elucidated, in vitro experiments and the *saa1/2* knock-out mice model demonstrated that SAA enrichment in HDL per se can impair CEC. As CRP is a known acute phase reaction reactant released by the liver along with SAA^31^, the impaired CEC and its relevance to hsCRP in ACS patients, as shown in our study, could possibly result from an increase of SAA content in HDL. ApoA-I modification could be another reason for decreased CEC. Our previous experimental study showed that myeloperoxidase (MPO)-mediated tryptophan oxidation of apoA-I was harmful to HDL-mediated cholesterol efflux^32^.

It has been widely reported that hsCRP is a valuable marker of CAD. For example, CAD patients with higher hsCRP baseline level (usually ≥ 2 mg/L) have a significantly higher risk of major adverse cardiovascular events (MACE) compared to those with lower hsCRP baseline level (usually < 2 mg/L)^33,34^. In the CANTOS trial, participants with canakinumab, a monoclonal antibody targeting interleukin-1β, who achieved an on-treatment hsCRP level < 2 mg/L had a 25% reduction in the risk of MACE, while no significant benefit was observed in participants whose on-treatment hsCRP concentration was 2 mg/L or above^35^. On the other hand, treatment with another anti-inflammation medicine, methotrexate, did not reduce the risk of MACE, as there was no decrease in hsCRP levels following treatment^36^. Based on these findings and the results of our study, the reason why hsCRP serve as a marker of CAD can be explained, at least partially, by its ability to reflect HDL dysfunction.

We also found that CAD patients with higher hsCRP levels had more large HDL particles (HDL1) and less small HDL particles (HDL4), consistent with the previous study, which analyzed HDL subclasses by electrophoresis^19,37^. In our study, the exact mechanism by which inflammation affects HDL remodeling remains to be further elucidated.

In conclusion, we found that HDL-C levels could not reflect HDL's functional status in patients with CAD, and the impaired correlation between HDL-C levels and CEC was possibly due to inflammation-induced HDL-subclass remodeling. We also found that the level of hsCRP was inversely associated with CEC, although the association would not reach significance after adjusting for multiple testing. These hypothesis-generating data suggested that hsCRP levels, a marker of acute inflammation, may associate with HDL dysfunction in ACS subjects. As a relatively small group of patients was taken into account and most of our MI patients were beyond the super-acute-phase (96 h), further studies with larger cohorts, including stable CAD patients, patients with super-acute-phase MI will need to be undertaken to further verify these conclusions. Besides, it is noteworthy that this is a hypothesis generating paper, from a study not designed specifically to test the correlation between hsCRP and CEC, and our conclusions still warrant further investigations.

## Supplementary Information

Supplementary Figure 1.

Supplementary Figure 2.

Supplementary Tables.

## Abbreviations

HDL: High-density lipoprotein
HDL-C: High density lipoprotein cholesterol
CETP: Cholesteryl ester transfer protein
RCT: Reverse cholesterol transport
CEC: Cholesterol efflux capacity
CAD: Coronary artery disease
hsCRP: High-sensitivity C-reactive protein
hsTnT: High-sensitivity Troponin T
MI: Myocardial infarction
HIV: Human immunodeficiency virus
NMR: Nuclear magnetic resonance
ACS: Acute coronary syndrome
TG: Triglycerides
TC: Total cholesterol
LDL-C: Low-density lipoprotein cholesterol
DMEM: Dulbecco's Modified Eagle's Medium
ABCA1: ATP binding cassette transporter A1
ACAT: Acyl-coenzyme A: cholesterol acyltransferase
NMR-HDL-C: High-density lipoprotein cholesterol measured by NMR spectroscopy
SPSS: Statistical Package for Social Sciences
HDL1: The largest HDL subclass defined by NMR spectroscopy
HDL4: The smallest HDL subclass defined by NMR spectroscopy
MPO: Myeloperoxidase
ApoA-I: Apolipoprotein A-I
ApoA-II: Apolipoprotein A-II
ApoB: Apolipoprotein B
ApoC-III: Apolipoprotein C-III
SAA: Serum amyloid A
MACE: Major adverse cardiovascular events
