# Supplementary figures and images for: High-sensitivity CRP may be a marker of HDL dysfunction and remodeling in patients with acute coronary syndrome
Source: Sci Rep. 2021 Jun 1;11:11444. doi: 10.1038/s41598-021-90638-0 (PMC8169928; doi:10.1038/s41598-021-90638-0)

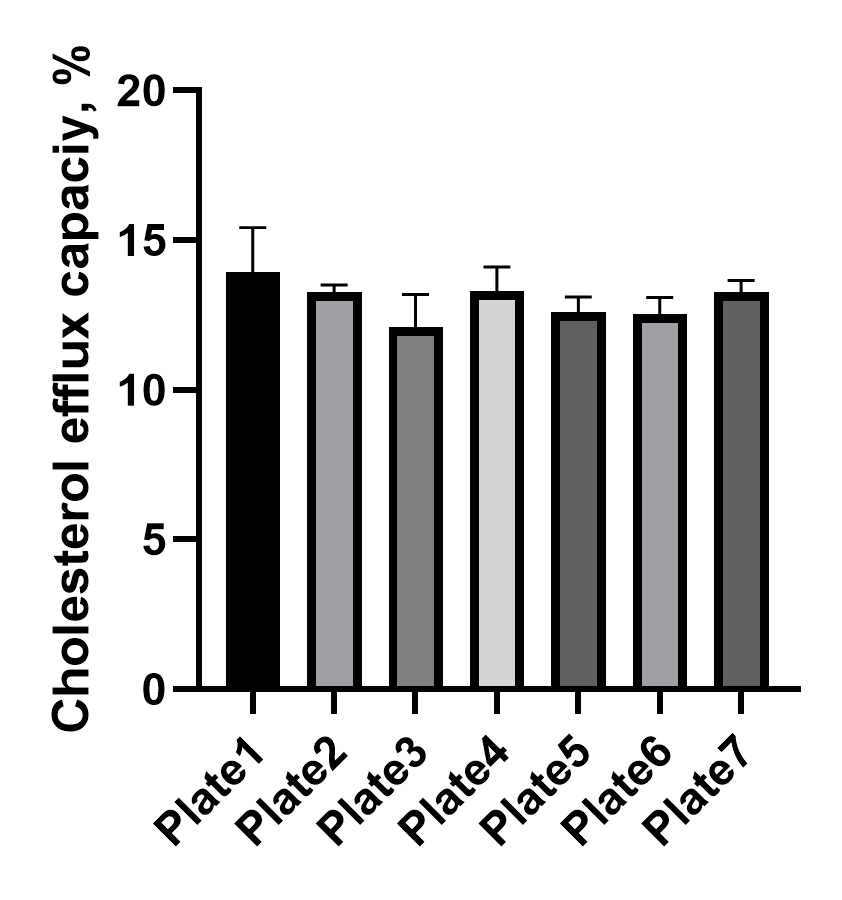

Supplement: Supplementary file 1 — Supplementary Figure 1. [file 41598_2021_90638_MOESM1_ESM.tif]

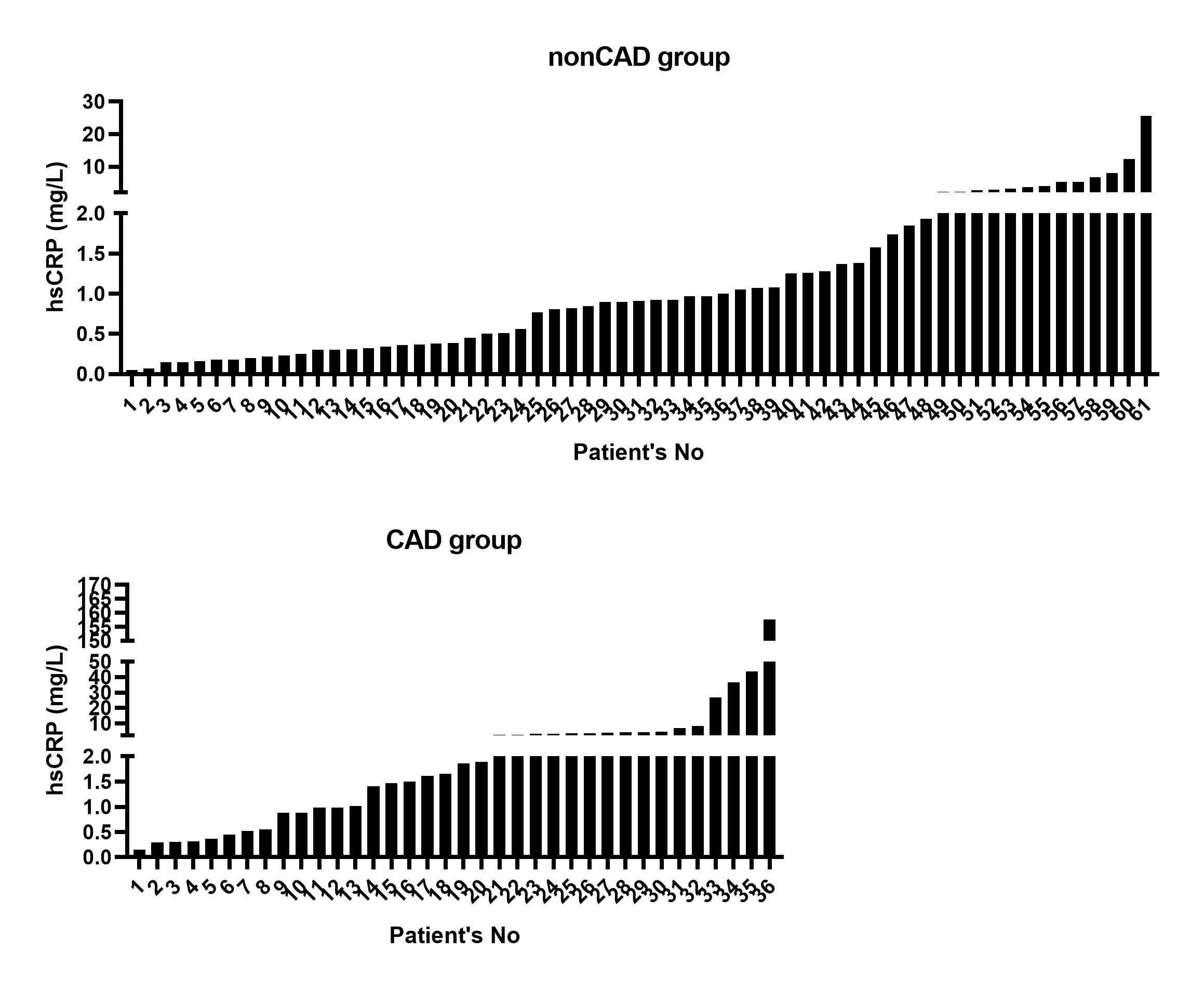

Supplement: Supplementary file 2 — Supplementary Figure 2. [file 41598_2021_90638_MOESM2_ESM.tif]
