# Supplementary material for: High-sensitivity CRP may be a marker of HDL dysfunction and remodeling in patients with acute coronary syndrome
Source: Sci Rep. 2021 Jun 1;11:11444. doi: 10.1038/s41598-021-90638-0 (PMC8169928; doi:10.1038/s41598-021-90638-0)
Supplement: Supplementary file 3 — Supplementary Tables. [file 41598_2021_90638_MOESM3_ESM.docx]

**Supplementary Table 1:** The number of CAD patients (n=36) with different onset time.

| The interval from illness onset to admission | Number of patients |
| --- | --- |
| <24h | 1 |
| (24h, 72h] | 1 |
| (3d, 7d] | 5 |
| (7d, 14d] | 3 |
| (14d, 1m] | 11 |
| (1m, 3m] | 5 |
| >3m | 10 |

CAD, coronary artery disease.

**Supplementary Table 2:** **Concentrations of NMR-determined total plasma apolipoprotein A-I-rich lipoprotein and 30 HDL-related lipoproteins in the non-CAD (n=61) group and the CAD (n=36) group.**

|  | | Non-CAD, mg/dL | CAD, mg/dL |
| --- | --- | --- | --- |
| *Total plasma apoA-I* | | 134.91±17.18 | 123.01±17.12^**^ |
|  | Cholesterol | 50.90±9.79 | 45.74±7.42^**^ |
|  | Triglycerides | 11.44±3.88 | 11.47±4.04 |
| *Total-HDL* | Phospholipids | 69.65±12.42 | 59.97±11.57^***^ |
|  | Free cholesterol | 10.39±2.86 | 8.43±2.32^**^ |
|  | ApoA-I | 132.85±17.98 | 120.91±18.61^**^ |
|  | ApoA-II | 28.69±3.53 | 26.88±4.09^*^ |
|  | Cholesterol | 15.42±5.93 | 13.94±4.24 |
|  | Triglycerides | 3.83±1.83 | 3.89±1.97 |
| *HDL1* | Phospholipids | 17.90±7.48 | 15.31±5.47 |
|  | Free cholesterol | 3.06±1.38 | 2.04±0.80^***^ |
|  | ApoA-I | 21.59±11.09 | 17.97±6.67 |
|  | ApoA-II | 1.74±1.16 | 1.35±1.18 |
|  | Cholesterol | 7.42±2.29 | 6.24±1.76^**^ |
|  | Triglycerides | 1.94±0.77 | 1.96±0.85 |
| *HDL2* | Phospholipids | 11.42±3.53 | 9.75±3.34^*^ |
|  | Free cholesterol | 1.21±0.58 | 0.70±0.48^***^ |
|  | ApoA-I | 15.38±3.78 | 14.20±3.96 |
|  | ApoA-II | 2.47±0.99 | 2.18±1.33 |
|  | Cholesterol | 8.71±1.65 | 7.20±1.94^***^ |
|  | Triglycerides | 2.23±0.82 | 2.26±0.90 |
| *HDL3* | Phospholipids | 13.97±2.72 | 11.68±3.46^***^ |
|  | Free cholesterol | 1.34±0.56 | 0.81±0.58^***^ |
|  | ApoA-I | 23.50±4.40 | 20.94±5.47^*^ |
|  | ApoA-II | 5.06±1.14 | 4.32±1.63^*^ |
|  | Cholesterol | 18.30±4.12 | 17.06±5.36 |
|  | Triglycerides | 3.72±1.07 | 3.92±0.83 |
| *HDL4* | Phospholipids | 25.25±4.42 | 22.75±6.20^*^ |
|  | Free cholesterol | 2.95±1.13 | 2.30±1.21^**^ |
|  | ApoA-I | 70.43±11.80 | 66.98±15.17 |
|  | ApoA-II | 17.76±3.69 | 16.98±3.71 |

NMR, nuclear magnetic resonance; CAD, coronary artery disease; HDL, high density lipoprotein; ApoA-I, apolipoprotein A-I; ApoA-II, apolipoprotein A-II. ^*^p<0.05, ^**^p<0.01, ^***^p<0.001.

**Supplementary Table 3:** **Pearson’s correlation analysis between CEC and clinical parameters in the non-CAD (n=61) group and the CAD (n=36) group.**

|  | Non-CAD | | CAD | |
| --- | --- | --- | --- | --- |
|  | r | p | r | p |
| Age, y | 0.119 | 0.374 | -0.075 | 0.665 |
| BMI, kg/m^2^ | 0.056 | 0.680 | -0.181 | 0.321 |
| Gensini score | NA | NA | 0.074 | 0.673 |
| hsTnT, µg/L (log-transformed) | NA | NA | -0.179 | 0.092 |
| Total cholesterol, mg/dL | 0.288 | 0.025 | 0.114 | 0.508 |
| Triglycerides, mg/dL (log-transformed) | 0.031 | 0.813 | -0.102 | 0.552 |
| LDL-C, mg/dL | 0.217 | 0.093 | 0.073 | 0.673 |

CEC, cholesterol efflux capacity; CAD, coronary artery disease; BMI, body mass index; LDL-C, low density lipoprotein cholesterol; NA, not applicable.

**Supplementary Table 4: Comparison of baseline characteristics and NMR-determined total HDL-related lipoproteins between hsCRP-low CAD patients (n=18) and hsCRP-high CAD patients (n=18).**

|  | hsCRP-low | hsCRP-high | p |
| --- | --- | --- | --- |
| Age, y | 60.2±8.5 | 61.4±8.9 | 0.663 |
| Male sex, n (%) | 15 (83.8) | 14 (77.8) | 0.674 |
| BMI, kg/m^2^ | 25.0±3.0 | 24.5±3.3 | 0.678 |
| Diabetes, n (%) | 4 (22.2) | 8 (44.4) | 0.157 |
| Hypertension, n (%) | 9 (50.0) | 8 (44.4) | 0.738 |
| Statin use, n (%) | 11 (61.1) | 16 (88.9) | 0.054 |
| Current smoking, n (%) | 8 (44.4) | 7 (38.9) | 0.735 |
| Drinking, n (%) | 4 (22.2) | 3 (16.7) | 0.674 |
| hsTnT, µg/L | 0.0130 (0.0077-0.0283) | 0.0815 (0.0084-0.3994) | **0.031** |
| *Chemical-determined lipids* | | | |
| Total cholesterol, mg/dL | 150.28±37.11 | 146.07±37.62 | 0.737 |
| Triglycerides, mg/dL | 171.83 (97.65-237.37) | 159.87 (100.08-234.05) | 0.888 |
| HDL-C, mg/dL | 37.94±8.76 | 34.14±8.96 | 0.207 |
| LDL-C, mg/dL | 90.42±31.87 | 89.05±32.26 | 0.898 |
| *NMR-determined lipids and apolipoproteins* | | | |
| NMR-HDL-C, mg/dL | 45.99±7.39 | 45.49±7.66 | 0.844 |
| Total-HDL-phospholipids, mg/dL | 60.75±14.02 | 59.20±8.82 | 0.695 |
| Total-HDL-triglycerides, mg/dL | 11.62±4.38 | 11.31±3.79 | 0.826 |
| Total-HDL-free cholesterol, mg/dL | 8.36±2.50 | 8.51±2.20 | 0.842 |
| Total-HDL-apoA-I, mg/dL | 123.87±21.56 | 117.95±15.17 | 0.347 |
| Total-HDL-apoA-II, mg/dL | 27.68±4.73 | 26.08±3.28 | 0.245 |

NMR, nuclear magnetic resonance; HDL, high-density lipoprotein; hsCRP, high-sensitivity C-reactive protein; CAD, coronary artery disease; BMI, body mass index; LDL-C, low-density lipoprotein cholesterol; hsTnT, high-sensitivity Troponin T; NMR-HDL-C, NMR-determined high-density lipoprotein cholesterol; ApoA-I, apolipoprotein A-I; ApoA-II, apolipoprotein A-II.

**Supplementary Table 5: Pearson’s correlation analysis between the levels of hsCRP and the concentrations of lipoproteins belonged to HDL1 subclass or HDL4 subclass in CAD patients.**

|  | HDL1 subclass | | HDL4 subclass | |
| --- | --- | --- | --- | --- |
|  | r | p | r | p |
| Cholesterol, mg/dL | 0.461 | 0.005 | -0.334 | 0.046 |
| Phospholipids, mg/dL | 0.338 | 0.044 | -0.402 | 0.015 |
| Free cholesterol, mg/dL | 0.209 | 0.222 | -0.283 | 0.094 |
| Triglycerides, mg/dL | 0.177 | 0.303 | -0.204 | 0.233 |
| ApoA-I, mg/dL | 0.223 | 0.191 | -0.369 | 0.027 |
| ApoA-II, mg/dL | 0.331 | 0.049 | -0.334 | 0.047 |

hsCRP, high-sensitivity C-reactive protein; HDL, high density lipoprotein cholesterol; ApoA-I, apolipoprotein A-I; ApoA-II, apolipoprotein A-II.

**Supplementary Table 6:** Experimental Parameters of nuclear magnetic resonance spectroscopy.

| **Parameters** |  |
| --- | --- |
| Pulse program | noesygppr1d |
| Time domain | 98304 |
| Dummy scans | 4 |
| Scans | 32 |
| Sweep width | 30 ppm |
| Acquisition time | 2.726 s |
| Relaxation delay | 4 s |
| Receiver gain | 90.5 |
| Dwell time | 27.7 µs |
| Mixing time | 0.01 s |
| Line broadening | 0.3 Hz |
